# The role of CD24 in multiple myeloma tumorigenicity and effects of the microenvironment on its expression

**DOI:** 10.18632/oncotarget.27190

**Published:** 2019-09-10

**Authors:** Nechama Gilad, Hila Zukerman, Marjorie Pick, Moshe E. Gatt

**Affiliations:** ^1^ Department of Hematology, Sharett Institute, Hadassah Medical Organization, Kiryat Hadassah, Jerusalem, Israel; ^2^ Department of Chemistry and Biology, Hebrew University, Jerusalem, Israel; ^3^ Department of Biomedical Engineering, Technion Institute of Technology, Haifa, Israel

**Keywords:** multiple myeloma, CD24, microenvironment, cell cycle and apoptosis changes, tumorigenicity

## Abstract

Multiple myeloma (MM) is an incurable neoplasm characterized by infiltration of malignant plasma cells (PCs). Recently, the tumor microenvironment has become of great interest in MM as it known to be involved in progression and metastasis of the disease. CD24, is an adhesion molecule expressed during B cell maturation, is down regulated through the cells differentiation into PCs. Though the role of CD24 in solid cancers is well defined, its role in MM remains unknown. We aimed to understand the involvement of CD24 in MM by up-regulating its expression on MM cell lines by co-culturing the cells with bone marrow stromal cell (BMSCs). We then studied the differences between CD24+ and CD24− MM cells and found that CD24+ MM cells presented a less tumorigenic phenotype by impaired capability to migrate and to create colonies as compared with CD24− MM cells. Furthermore, there were significantly more apoptotic cells in the CD24+ fraction. Additionally, the CD24+ cells also upregulated CXCR4 expression. The decrease tumorigenicity correlated with a “more normal” PC immunophenotype in patients with MM and correlated with CD45 expression and a stronger expression of CXCR4. In summary, we found the expression of CD24 on PCs to correlate with attenuated tumorigenicity.

## INTRODUCTION

Plasma cells (PCs) are terminally differentiated B-cells [1, 2] that synthesize and secrete thousands of molecules of clonospecific antibodies in response to microbial pathogens [3]. They represent 0.25% of bone marrow (BM) mononuclear cells and can rarely be found in the peripheral blood (PB) [4]. Normal B cells development into PCs includes selection of B cells with high affinity to a specific antigen. The differentiation of B cells into a PC is a complex process and can result in PC disorders [2] including multiple myeloma (MM) and PC leukemia (PCL) [5–7] characterized by the expansion of clonal PC in the BM. MM can occur de novo but in most cases is preceded by a premalignant disease such as monoclonal gammopathy of undetermined signiðcance (MGUS) or smoldering MM (SMM) [8]. Despite several new drugs introduced in the recent years, MM remains incurable and the estimated median survival is seven years [9, 10]. Current therapeutic aims are to induce and maintain long-term remission [11].

Multiparameter flow cytometry (MFC) is an essential tool for the diagnosis, classification, prognosis and monitoring of MM. According to the European Myeloma Network diagnosis of MM by flow cytometry requires the use of complex of antigens, which allows distinction between normal, and aberrant PCs and can also indicate disease progression [12–14].

The BM milieu presents the microenvironment of the MM cells, and can contribute to the pathogenesis of MM cells and their ability to leave the microenvironment. Scientific evidence indicates that factors in the microenvironment affect PCs longevity and survival and can prolong the aberrant MM PCs lifespan and allow progression of the disease [15–17] depending on this complex interaction [18]. Accumulation of malignant PCs in the BM results in aberrant feedback regulatory loop between the PCs and the BM cells. Understanding the cellular and molecular mechanism holding the interaction between the cancer cells and their microenvironment can contribute to development of novel therapeutic strategies [19].

CD24 is usually found as a surface membrane protein that is stored in microvesicular bodies in the cytoplasm before arriving to the cell membrane [20]. Its expression is shown to be regulated at a post transcriptional level by controlled RNA stability [21]. The CD24 molecule consists of a small protein core with only 27 amino acids [22] that attaches to the membrane via a Glycosylphosphatidylinositol (GPI)-anchor. A broad range of cells express CD24 on their surface including hematopoietic, neural, muscular and epithelial cells [23]. Investigating the expression of CD24 in B cells showed that CD24 is expressed in pre-B lymphocytes remains expressed on mature resting B cells and becomes down-regulated during the maturation process to PCs [24, 25]. Other research indicated that CD24 not only correlated with the maturation of the B cells but is also involved in the activation and differentiation of the cells, as CD24-deficiency results in a decrease in late pre-B and immature B-cell populations in the BM [26]. CD24 is broadly over-expressed on many types of tumor tissues and indicates a correlation between its expression and metastasis and points at an active role for CD24 in this process in lung cancer [27], breast cancer [28] and prostatic cancer [29]. Furthermore, in several cancers, correlation between high expression of CD24 and shorter patient survival time has been found [29]. Thus, CD24 has become an important marker of diagnosis and prognosis for some solid cancers in recent years. Recently, CD24 mRNA has been shown to be downregulated in PCs of MM patients and correlated to worse overall survival [30] however, its function and the role of CD24 protein was not validated and is still unknown. In this study, we attempted to decipher the role of CD24 in MM PC clones, and to understand the effects the microenvironment may have on CD24 expression. Understanding the role of CD24 in MM cells may contribute to predict the course of MM in the individual patient, and may aid in the selection of a more specific treatment for patients. In addition, understanding the involvement of the microenvironment in CD24 up-regulation can help to unravel better drugs enable to increase CD24 expression in patient’s MM cells and decrease MM tumorigenicity.

## RESULTS

### CD24 protein is not expressed by multiple myeloma cell lines

Expression levels of CD24 in 7 different MM cell lines were assessed of which 3 are represented (Supplementary Figure 1). Surface and cytoplasmic expression of CD24 on the MM cell lines was analyzed and compared to two myeloid control cells lines: LAMA-84, with high CD24 expression, and HL60, with no CD24 expression (Supplementary Figure 1A). All MM cell lines tested did not express CD24 on their surface or cytoplasm (Supplementary Figure 1B). Two normal B cell lines were selected as phenotypic controls for the MM cell lines, SKW6 and 721.221, where 721.221 expressed higher levels of surface CD24 than SKW6 (Supplementary Figure 1).

We next tested CD24 mRNA expression in B and MM cell lines by quantitative Real Time-PCR (qRT-PCR). We found that 6 of 9 MM cell lines, and the 721.221 B cell line had high levels of CD24 mRNA relative to the protein positive LAMA-84 cell line (Supplementary Figure 2). Furthermore, high levels were found in the HL60 cell line. These results suggest that CD24 expression may be regulated at the post-transcription level.

### Bone marrow stromal cells generated from MM patients can up-regulate CD24 expression on MM lines

We hypothesized that the microenvironment could regulate CD24 expression on MM cells. Thus CD24 expression was analyzed after co-culturing the MM and B cells lines with BMSCs generated from MM patient’s BM samples at various time points of treatment. Two cell lines (KMS11 and JJN3) showed an up-regulation of CD24 expression after incubation with BMSCs (Supplementary Figure 3), and were further used for subsequent analysis of CD24 up-regulation over a period of time (Supplementary Figure 3A). For future experiments results are shown as the fold-increase of CD24 expression of cells incubated with BMSC over controls not incubated with BMSCs at day 4 (Supplementary Figure 3C). The absolute CD24 up-regulation on the MM cell lines after incubation on BMSC was always subtle and mimicked levels detected on normal PCs. The relative up-regulation was significant but not as an over-expression that was observed in solid tumors. To be sure that the CD24 expression observed was exclusively on the B and MM cells, once confluent and before incubation with MM and B cells, BMSC were trypsinized and assessed for CD24 expression by flow cytometry. Very little expression was detected as compared to Isotype control (Supplementary Figure 3D).

### CD24 affects MM clonogenicity and migration

**Figure 1:**
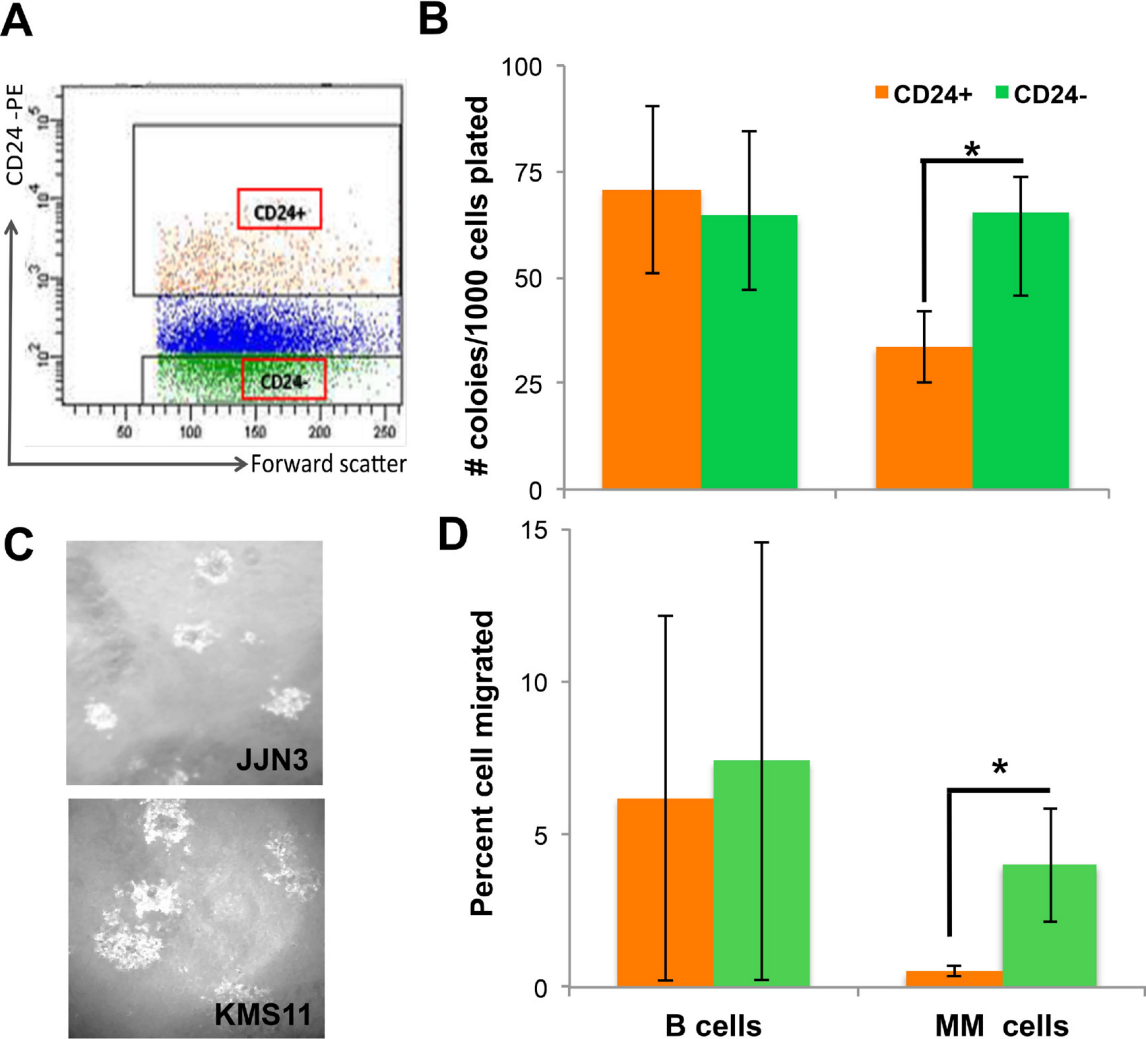
CD24 up-regulation on MM cells decreases tumorigenicity. (**A**) Representative dot plot of gating strategy for sorting experiments of MM cell line. CD24+ - orange and CD24- green dots represent the populations that were sorted (**B**) Number of colonies (mean ± SEM) generated in methylcellulose from 1000 sorted CD24+ (orange) and CD24- (green) cells. B cells (*n =* 10) or MM (*n =* 16) were incubated for 5 days on BMSC generated from MM patients BM. Collected, stained and sorted for CD24 expression. (**C**) A representative image of colonies generated from JJN3 and KMS11 CD24+ cells lines. (**D**) Percent (mean ± SEM) of sorted CD24+ (orange) and CD24- (green) cells that migrated from 50,000 cells seeded. B *n =* 5) or MM (*n =* 5) cells were placed in the upper well of a two chamber Transwell incubated overnight to allow cells to migrate towards high fetal calf serum containing medium. ^*^(*P <* 0.05).

CD24 up-regulated cells were sorted, and two fractions were collected (Figure 1A); Sorted CD24+ and CD24- fractions were seeded and the colonies formed from the two populations showed that CD24+ MM cells generated significantly fewer colonies than the CD24- population (*P <* 0.04); while in B cells this difference was not observed (Figure 1B). The colonies generated by JJN3 and KMS11 MM cell lines were similar in morphology and results were combines for all experiments and re-named “MM cells lines” (Figure 1C). Within the control B cells line used- SKW6 and 721.221 results were similar between the two lines and were combines and labeled as “B cells” for future experiments.

To further evaluate the effect of CD24 up-regulation a migration assay was performed on the sorted CD24+ and CD24- populations from both MM and B cells lines. As shown, CD24+ MM cells scarcely migrated as compared with the CD24- MM cells (*p* = 0.04). This in a MM specific manner, in contrast to the control B cells which showed no differences (Figure 1D). Taken together, these results demonstrates that MM cells with normal surface expression of CD24 seen on normal PCs and induced by incubation with BMSC, exhibit lower colony formation and migration (assays assessing tumorigenicity) compared with their low-CD24 counterparts. This effect is unique to MM cells and is not observed when comparing CD24-high and CD24-low B cells (Figure 1).

### Evaluation of apoptosis by cell cycle

**Figure 2:**
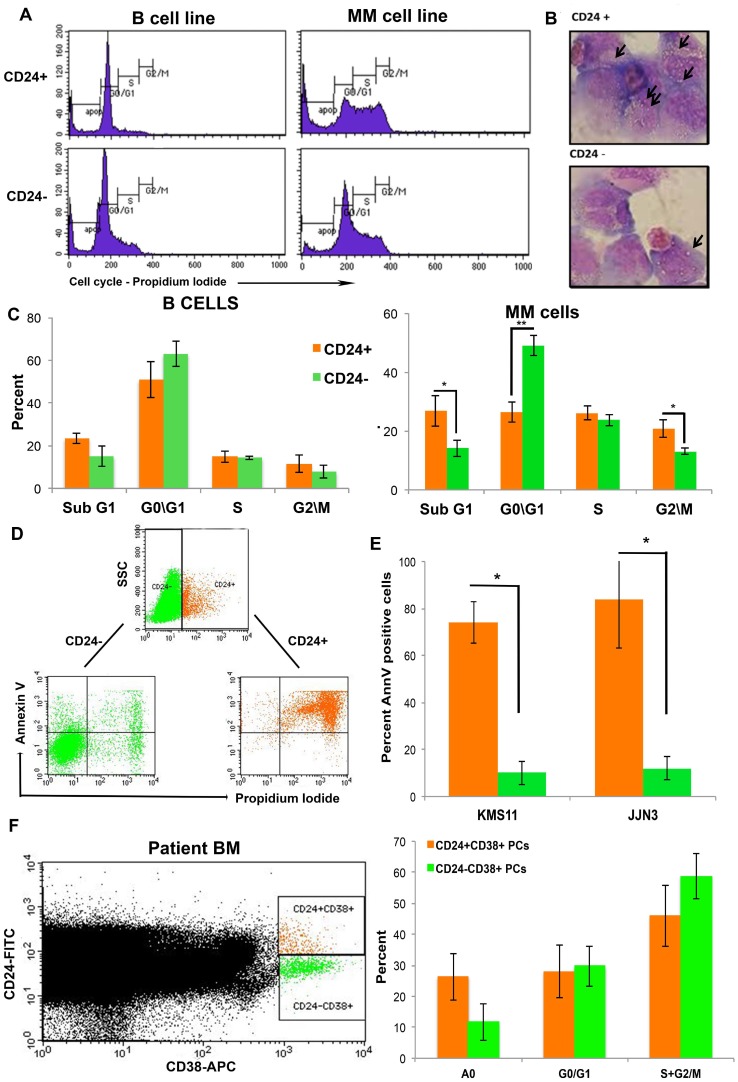
Up-regulation in CD24 expression correlates with increased apoptosis in MM cells. (**A**) Representative histograms of 5 repeated experiments of cell cycle analysis of sorted CD24+ and CD24- B and MM cells (**B**). Representative image of the morphology of CD24+ and CD24- MM cells after May-Grumwald/Giemsa staining. Arrows point to vacuoles Note the increase in vacuoles in the CD424+ cells (magnification 100x) and (**C**) Quantitation of cell cycle results (mean ± SEM, *n =* 7) (**D**). Representative dot plot analysis of Annexin V and PI staining in MM cells (KMS11) after incubation for 5 days with BMSC from patient with MM. The cells were gated as CD24+ (orange) and CD24- (green) and analyzed for Annexin V/ PI. (**E**) Summary of the percent (mean ± SEM, *n =* 3) Annexin V+ cells in CD24+ (orange) and CD24- (green) gated fraction of MM (KMS11 and JJN3). (**F**) Representative dot plot of CD38+CD24+ (orange) and CD38+CD24- (Green) PCs in patients with multiple myeloma were assessed for percent apoptosis and cell cycle (*n =* 9, ^*^ *P <* 0.04)

Upon cross-linking and activation, CD24 is known to be involved in inducing apoptosis in immature B cells [31]. Indeed, cell cycle analysis showed a significant increase in the percentage of cells in SubG1 apoptotic area (*p* = 0.04) in the CD24+ MM cells and decreased percentage of cells in G0/G1 area (*p* = 6.27E-05) as compared with the CD24- MM cells (Figure 2A and 2C). These differences were not observed in the control sorted B cell lines (Figure 2A and 2C). The CD24+ MM cells had more vacuoles in their cytoplasm compared with the CD24- cells- reminiscent of apoptosis (Figure 2B) [32]. No differences were seen in the control sorted B cell lines’ morphology (data not shown). Finally, Annexin V staining show a significant increase in apoptotic cells in the CD24+ fraction as compared with the CD24- sorted cells (*P <* 0.05) (Figure 2D and 2E). This indicates a strong correlation between CD24 expression and cell death by apoptosis.

### Cells undergoing apoptosis do not up-regulate CD24 expression

Our previous results indicate a correlation between CD24 up-regulation and increased apoptosis in MM cells. To further show that CD24 is not a mere surface marker of apoptosis, and that the apoptosis is secondary to the CD24+ phenotype and not vice- versa, we incubated the cells with a compound known to induce apoptosis in MM cells. Velcade is a proteasome inhibitor that inhibits protein degradation and leads to cells apoptosis [33].

JJN3 MM cells were treated with 10, 20 and 40 nm Velcade for 48 hours and stained for Annexin V, propidium iodide together with CD24. After 48 hours of incubation with Velcade Annexin V and propidium iodide positive cells were detected, however no CD24 up-regulation was observed (Supplementary Figure 4). The double positive Annexin V/Propidium iodide cells were a significantly increase in percent (Supplementary Figure 4). The same experiment was performed with etoposide, a chemotherapy antibiotic inducing apoptosis, with the same effects [not shown]. These results suggest that the apoptotic process does not cause up-regulation of CD24 in MM cells, and CD24 is not a surrogate marker for apoptosis.

### CD24+CD38+ Plasma cells in patient samples are more apoptotic

To confirm our results, patients’ samples were assessed by flow cytometry for the expression of surface CD24 and CD38. Cell cycle analysis of the CD24+CD38+ and CD24-CD38+ PCs showed a significant increase in apoptosis in the CD24+ versus the CD24- fraction of PCs (Figure 2F, *P <* 0.04, *n =* 9). This analysis further validates the previously described *in vivo* results.

### Direct interaction with the BMSC is important for CD24 up-regulation on MM cells

**Figure 3:**
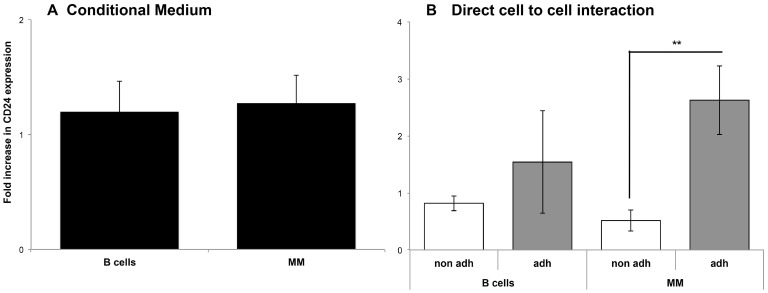
Direct interaction with the BMSC is necessary for CD24 up-regulation in MM and B cells. (**A**) Fold increase of CD24 expression compared to control cells not incubated with BMSC in B (*n =* 9) and MM (*n =* 18) cells incubated with conditional medium over cells incubated with regular medium as a control for 5 days. CD24 expression was analyzed by flow cytometry. (**B**) Fold increase in CD24 expression compared to control cells not incubated with BMSC in the adherent and non-adhered fractions from B (*n =* 5) and MM cells incubated with BMSC for 5 days (*n =* 11) ^**^(*P <* 0.01) (mean ± SEM).

To investigate if the CD24 up-regulation observed is due to direct contact with BMSCs or due to paracrine secretion of growth factors, condition-medium (CM) from confluent BMSCs cultures was seeded with MM and B cells. The CD24 expression levels where not significantly up-regulated in both MM and the B cell lines when CM from BMSCs was mixed with the cells (Figure 3A). We observed in the previous experiments, that the incubation of MM and B cells with patient’s BMSCs, produced a fraction of cells that adhered to the BMSCs and a fraction that remained suspended in the medium. In previous experiments we collected all the MM and B cells, after incubation with BMSCs, for measurement of CD24 up-regulation. Since it wasn’t the CM that effected CD24 expression on the MM cells we wanted to assess if direct contact with BMSC was essential. To this end we collected the two fractions separately: the ‘adherent’ cells and cells that remained in the supernatant or ‘non-adherent’ cells. These two fractions were collected, stained and analyzed by flow cytometry (refer to supplementary methods). We observed a significant up-regulation of the CD24 expression in the MM cells in the ‘adherent’ compartment as compared with ‘non-adherent” cells (*p* = 0.007), not seen in the B cells (Figure 3B). Thus direct interaction with BMSCs microenvironment is essential for up-regulation of CD24 expression on MM cell lines, in a MM-specific and unique process.

### Testing possible effect of CD24 up-regulation on CXCR4 expression

**Figure 4:**
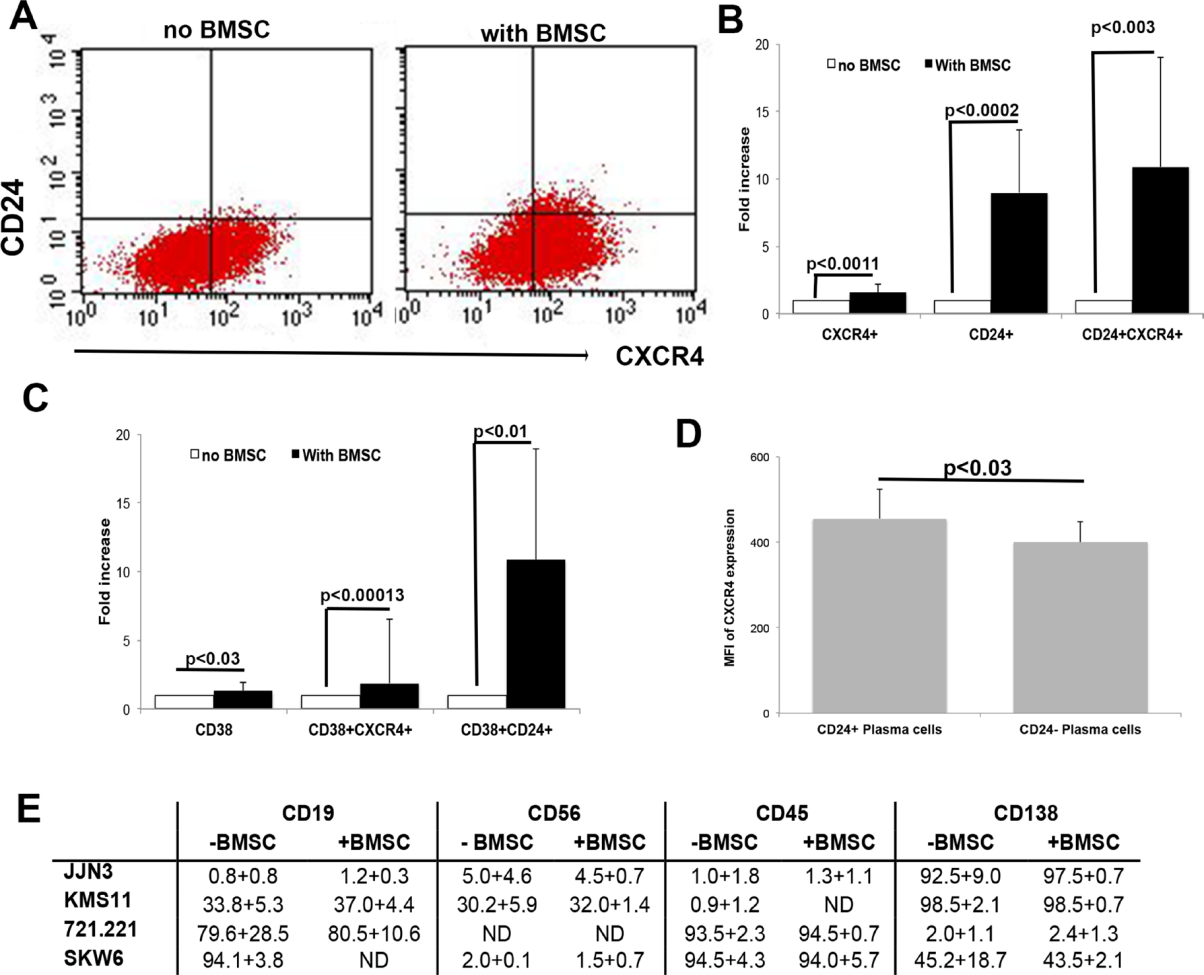
Up-regulation of CD24 correlates with CXCR4 and CD38 expression in MM cells. (**A**) Representative dot plots of CD24 and CXCR4 expression before and after incubation with BMSCs. (**B**) Fold increase in expression of CXCR4, CD24 and double positive CXCR4/CD24 cells after 5 days incubation of MM cells with BMSC of patients with MM. Fold increase was significant (*p* < 0.0011–0.0002, *n =* 8). (**C**) Fold increase in expression of CD38, double positive CD38/CXCR4 and CD38/CD24 cells after 5 days incubation of MM cells with BMSC of patients with MM. Fold increase was significant (*P <* 0.03–0.0013, *n =* 8). (**D**) Mean fluorescence intensity (MFI) of CXCR4 expression on CD24 positive (+) and CD24 negative (-) plasma cells of multiple myeloma patients. (*P <* 0.03, *n =* 10). (**E**) Changes in expression of other markers were assessed. No significant upregulation was detected (*n =* 5)

The chemokine receptor CXCR4 is involved in cell invasion and proliferation in many tumors including MM [34]. MM and B cell lines were assessed for changes in CD24 and CXCR4 expression after incubation with BMSCs. Robust levels of CXCR4 were found on the MM cell lines (Figure 4A), however, after incubation with BMSCs from patients, this expression was further increased significantly by1.57 ± 0.6 fold (*n =* 8, *P <* 0.011, Figure 4A and 4B, total CXCR4). Furthermore, the double positive CD24+CXCR4+ fraction significantly increased after incubation with BMSCs with a mean fold expansion of 10.89±8.11 (*n =* 8, *P <* 0.003, Figure 4A and 4B). Thus it was the population of MM cells that upregulated CD24 that also upregulated CXCR4 expression.

### Changes in other plasma cell markers correlates with CD24 up-regulation

To rule out a possibility that the CD24 up-regulation as an adhesion molecule is a bystander effect of co-culturing the cells with BMSCs, we assessed changes in expression of other significant surface markers found on MM PCs: CD38, CD138, CD38, CD45, CD19 and CD56 after incubation. Both CD45 and CD38 are adhesion molecules. No significant up-regulation on MM cells was observed for CD138, CD45, CD19 or CD56 by the BMSCs (Figure 4E). However, we did observe a significant up-regulation of CD38 after incubation with BMSCs (*P <* 0.03, *n =* 8, Figure 4C). CD38 is expressed on most bone marrow leukocytes but is highly expressed on PCs and is used as the primary identifier of PCs in the BM, Is has been found to decrease in intensity on malignant MM PCs [7]. CD38 up-regulation correlated with CXCR4 up-regulation and there was a significant increase in this double positive CD38+CXCR4+ population after incubation on BMSCs (*P <* 0.0013, *n =* 8, Figure 4B). Furthermore, up-regulation of CD38 coincided with up-regulation in CD24 on MM cells (*P <* 0.01, *n =* 8, Figure 4C). To validate our finding *in vivo*, we analyzed BM samples of MM patients, and characterized their PCs for CXCR4 expression and found that all PCs expressed CXCR4 (not shown). However, the intensity of CXCR4 (Mean fluorescent intensity) expression was significantly higher in the CD24+ PCs as compared to the CD24- PCs (*P <* 0.03, *n =* 10, Figure 4D). We therefore believe that part of the role of CD24 up-regulation is to control the CXCR4 surface expression.

### MM disease progression modifies the ability of BMSCs to up-regulate CD24 on MM cell lines

**Figure 5:**
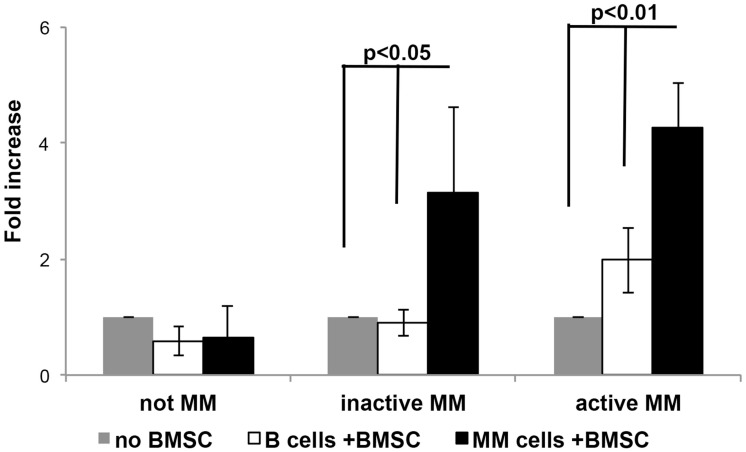
The effect of MM progression on CD24 up-regulation on MM and B cell lines. Fold increase in CD24 expression in B (Not MM *n =* 3, Inactive MM *n =* 6 and Active MM *n =* 16) or MM (Not MM *n =* 6, Inactive MM *n =* 10 and Active MM *n =* 31) cells incubated with BMSCs over B or MM cells incubated without BMSCs. (*P <* 0.01 - 0.05, mean ± SEM).

The CD24 up-regulation was observed upon incubation of MM and to a lesser degree B cell lines with BMSCs generated from patients’ BM at diverse stages of their disease. Patient samples were divided into the following MM stages: inactive asymptomatic MM (MGUS, SMM and remission), active MM (diagnosis, relapse and resistant), and as a control, we generated normal BMSCs (Not MM). Results show that up-regulation of CD24 expression on MM cells was more robust than on the B cells, after incubation with BMSC generated from patient samples (Figure 5). A significant difference in the up-regulation in CD24 expression was observed when the MM cells were co-cultured with BMSCs from patients with inactive (*p* = 0.05) and active (*p* = 0.01) disease (Figure 5). However, no increases in CD24 expression were observed in the B cells when incubated inactive MM BMSCs, and insignificant increase when active MM BMSCs were used (Figure 5). BMSCs generated from normal BMs (Not MM) did not increase CD24 expression neither on MM nor on B cells (Figure 5). To determine if the BMSC from patients in remission after treatment could increase CD24 expression in comparison to patients with MGUS or SMM (inactive MM) was further explored, yet no significant differences were observed when this patient group was separated (Supplementary Figure 5).

### CD24 expression correlates with a “normal” immunophenotype of plasma cells in patients with multiple myeloma

**Figure 6:**
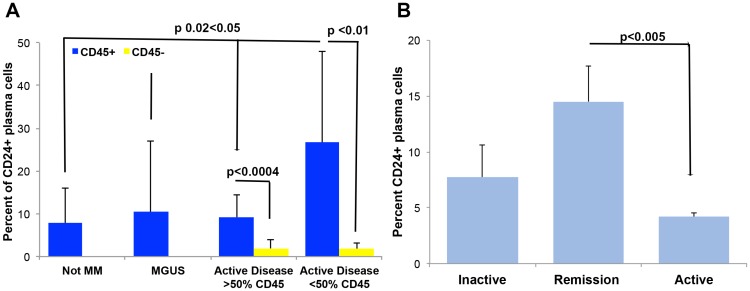
CD24 correlates to a more normal plasma cell. (**A**) CD24 expression was assessed on plasma cells from patients with no disease (Not MM *n =* 20), MGUS patients (*n =* 37), MM patients with more than 50% of PCs expressing CD45 (*n =* 12) and MM patients with less than 50% of PCs expressing CD45 (*n =* 10). CD24 expression was similar in the normal, MGUS and patients with majority of normal PCs (>50% of PCs express CD45) whereas patients with mostly aberrant (<50% of PCs expression CD45) the CD45+ PCs expressed significantly higher percent of CD24. Additionally, CD24 expression is significantly higher on CD45+ plasma cells as compared to on CD45 negative plasma cells in samples of MM patients. PCs from patients' BM samples were gated on the basis of CD38 and then sub gated on CD45 expression. The percent of CD24 expression was then quantitated on both the CD45+ and CD45- plasma cells. (*P <* 0.01, *n =* 10, mean ± SEM). (**B**) Percent of CD24 expression on PCs from patients with inactive (MGUS, SMM, *n =* 4), remission (*n =* 3) and active MM (Diagnosis, Relapse, *n =* 16) was assessed. Significantly more CD24 positive cells were found on the plasma cells of patients in remission as compared to active MM (*P <* 0.005, mean ± SEM).

CD45 expression characterizes the normal PC immunophenotype, and the majority of patients with MM have very little or no expression of CD45 on their PCs in the BM [7]. Since *in vitro* CD24 up-regulation results in a decreased tumorigenicity of MM cells we speculated that patients harboring a normal CD45 expression on their PCs might also express more CD24. We analyzed primary BM samples of normal, MGUS and MM patients. We divided the MM patients into two groups: 1. Greater than 50% CD45+ expression on the PCs and 2. Less than 50% CD45+ expression on the PCs. Patients with less than 50% PCs expressing CD45 had a significantly higher percentage of CD24 on the remaining CD45+ PCs (*p* ≤ 0.05, Figure 6A). Furthermore, the patients with active disease the CD45+ plasma cells expressed a significantly higher percentage of CD24 than CD45- plasma cells (*p* ≤ 0.01, Figure 6A) irrespective of CD24% expression on the CD45+ PCs. These results are consistent with our *in vitro* results and together suggest another correlation between CD24 expression and decreased tumorigenicity in MM cells.

To further validate our *in vitro* results, we analyzed the expression levels of CD24 in correlation with MM disease state (inactive, remission versus. active) in primary PCs of BM samples from MM patients. We found that the PCs (whether aberrant or normal) analyzed in patients with inactive and active MM expressed similar levels of CD24 however, MM cells of patients in remission after chemotherapy treatment, showed significantly higher CD24 expression on the PCs since these patients have less or no aberrant PCs a more normal CD45+CD19+CD56- immunophenotype is observed (Figure 6B, *p* < 0.05). These results strengthen our observation that CD24+ expression is a marker of less aggressive PCs in MM.

## DISCUSSION

CD24 is expressed in a broad range of cells [23]. Isolation of CD24 from different tissues or cell types presented a broad range of molecular weights and attributed to a high variability in CD24 glycosylation [35]. The many glycosylation sites of CD24 enable the diverse structure of the molecule in different cell types and in BMSC that we generated we were not able to detect the p-selectin ligand found in most solid tumors (data not shown). In B cells, the expression of CD24 becomes down regulated during the maturation process to PCs where its expression is low [24]. Studies of CD24 indicated that its over-expression affected the metastatic tumor spread and was associated with a more aggressive disease in many tumor types [36, 37], and is used as diagnostic marker in solid tumors [27]. Its role in MM remains unknown. Thus, we hypothesized that CD24 plays a role in MM aggressiveness. We found that all the MM cell lines examined did not express CD24 (Supplementary Figure 1A) yet by qRT-PCR studies we found robust levels of CD24 mRNA in 7 of the 10 MM cell lines tested (Supplementary Figure 2). Indeed, previous studies show that although CD24 antigens are lowly expressed on primary MM PCs, gene expression profiling revealed that CD24 RNA expression plays a significant role in MM prognosis [30].

The MM cell lines utilized in this project originate from patients’ PCL where the cells represent a more aggressive phenotype and no longer depend on the BM microenvironment to grow and survive. We assumed that this partly explains why all the MM cell lines tested were CD24 negative, while primary MM PCs detected in patients, especially when they were in remission, presented with CD24 expression (Figure 6). To try and understand this discrepancy we generated a BMSC microenvironment from patients BM samples and then incubated MM and B cells from cell lines to induce CD24 expression. Using this co-culture method, we were able to up-regulate the CD24 levels similar to the slight expression of CD24 on primary normal PCs (Supplementary Figure 3). Furthermore, this method does not involve any genetic manipulations unnaturally affecting the cell phenotype. In order to evaluate the effect of CD24 up-regulation on MM and B cells after incubation with BMSC we sorted the two fractions (Figure 1A) and studied colony formation, migration and cell death. CD24+ MM cells generated significantly fewer colonies than CD24- MM cells, migrated less (Figure 1) and where more apoptotic (Figure 2), which suggests a correlation between mild CD24 up-regulation and decreased tumorigenicity of the CD24+ MM cells. In these assays the normal B cell lines used as controls showed no differences in colony formation and ability to migration, whether CD24 was upregulated or not (Figures 1 and 2), attesting for the specificity of our finding to the MM cells. Although metastatic process is attributed mainly to solid cancers, hematological or ‘liquid’ tumors can also present with similar features. In MM, PCs are liable to undergo epithelial to mesenchymal transformation (EMT)-like process resulting in migration of the cells to other BM area and even generate extra-medullary disease [34, 38]. Our results demonstrate that CD24+ MM cells have impaired migration capability as compared with CD24− cells and thus could affect their ability to migrate out of the BM niche. This finding is in contrast to solid tumors where the high over-expression, rather then down-regulation, of CD24 is associated with a more aggressive phenotype. The involvement of CD24 in solid tumors and the metastatic process is attributed mainly to its interaction with the P-Selectin ligand presented on activated endothelial cells and platelets [36]. In B cells, CD24 shows no such interactions with the P-selectin ligand [27] and may explain the distinction of myeloma from solid tumors.

Several studies have shown that cross-linking of surface CD24 can cause DNA damage-induced apoptosis [39] and this might explain why the upregulated MM cells underwent apoptosis (Sub G0 peak, Figure 2), again specific for MM cells only. We believe this discrepancy might be due to the B cell lines being more immature, and express robust CD24 levels. Indeed, previous studies have shown that the cross-linking of CD24 generates different responses in the B cells depending on their stage of maturation [31]. In addition, the expression of CD24 is essential in pre-mature B cells, but its down-regulation is a required process in order to enable differentiation and maturation of the normal B cells [31]. These findings were validated by the morphological examination of sorted CD24+ and CD24− MM cells which showed clear vacuoles in the CD24+ MM cells (Figure 2B) a phenomenon which occurs in apoptosis [32].

In the last decade, many studies have been devoted to understanding the role of the tumor microenvironment and plays a critical role in cancer progression and metastasis [19]., also in MM [40]. Furthermore, the microenvironment contributes to EMT and lytic lesions by allowing the MM cells to migrate and home to other sites [8, 41]. We found that the up-regulation of CD24 by BMSCs did not involve any factors secreted by the microenvironment, as no such up-regulation was found by incubating the cells with BMSC-derived condition media (Figure 3A). Instead, we observed in the MM cell lines that up-regulated CD24 occurred when there was direct interaction with the BMSCs during the incubation (Figure 3B). Further studies are required to determine what protein/s on the BMSC are involved in this interaction

Both the MM cells and the BM microenvironment undergo changes upon MM progression [42]. To further assess the effect of the MM progression on CD24 expression we categorized the BMSCs collected from MM patients into two main groups according to the disease state; inactive (MGUS, SMM and complete remission patients) and active (diagnosis, relapse and resistant patients) MM. BMSCs generated from MM patients, either with active or inactive disease, were able to up-regulate CD24 expression, whereas normal BMSCs (control) were unable to up-regulate CD24 (Figure 5B and Supplementary Figure 3B). These results may indicate that the CD24 surface protein of MM cells can be up-regulated only by some BMSCs generated from patients suffering from MM, and that there is a specific and unique interaction that is responsible for CD24 regulation on MM cells and are microenvironment dependent. This observation might suggest a less aberrant and more “normal” microenvironment in these selective patient’s BMSCs that could possibly be an advantage in these patients’ long-term survival.

On further analysis of clinical results of patient samples that were assessed in the department we found that 1) CD45+ PCs contained a significantly higher percentage of CD24 expression than CD45− PCs the former being a more “normal” immunophenotype and 2) that the patient’s samples that were in remission had a significantly higher percentage of CD24+ PCs, than active MM samples (Figure 6). These results reinforce our *in vitro* result regarding the correlation between CD24 expression and decreased tumorigenicity, and that the slight up-regulation of CD24 expression on MM cells is associated with a less tumorigenic phenotype, and can be regulated by BMSCs. We believe have a more “normal” CD24 expression on PCs could be a good prognostic factor for MM patients and allow treatments to be more effective in these patients. If “normal” CD24 expression makes PCs more sensitive to apoptosis maybe the PCs will be more sensitive to various MM treatments, which induce apoptosis in patients and allow this group to have longer disease free survival. Future studies are needed.

In an attempt to understand the mechanism by which up-regulation of CD24 may affect MM cells migration and proliferation, we searched for possible interacting molecules with CD24. The chemokine receptor CXCR4 is involved in cell invasion and proliferation in many tumors and has been shown to promote the EMT like phenotype in MM cells [34]. Considering our results showing that BMSCs, which up-regulate CD24 expression on MM cells, causing impaired migration and colony formation, we speculated that these might be due to changes in CXCR4 expression. The co-culturing of MM cell lines with BMSC induced robust CXCR4 up-regulation together with CD24 and CD38. However, it was the double positive CD24/CXCR4 and CD38/CD24 population that was significantly increased, and not the single CXCR4+, CD38+ or double CD38+CXCR4+ populations (Figure 4B and 4D). Schabath *et al*. in 2006 demonstrated that CD24 disrupts CXCR4 function in pre-B cells by modifying the component in the lipid raft required for CXCR4 in order to induce signal transduction [43]. These findings explain how the MM cell lines that express CXCR4 together with CD24 did not migrate or generate colonies. There is some preliminary evidence from clinical studies that show high CXCR4 expression on PCs in MM correlate with a good prognosis [44] and we found that CXCR4 expression in primary CD24+ MM PCs from patient BM samples had a slight, yet significant increase in CXCR4 expression then CD24- PCs from the same patients (Figure 4C). Thus, we believe there is an interaction between CD24 and CXCR4.

In conclusion, we show that a the slight up-regulation of CD24 both on PCs *in vitro*, and validated in MM patient’s PCs, correlate to a less tumorigenic phenotype, that involves CXCR4 and CD38 co-expression. Our co-culture system proves that the microenvironment has a crucial role in PC regulation in MM disease. Further studies involving novel drugs that change the disrupted microenvironment effects through CD24 overexpression in MM may be a key for finding a successful therapy.

## METHODS

### Cell lines

MM cell lines: All cell lines were grown and maintained in RPMI media supplemented with 10% Fetal Calf Serum and incubated at 37° C, 5% CO_2_.

### Generation of bone marrow stromal cells from patient’s BM samples and co-cultures

BMSC have been generated from BM samples and all patients signed an informed consent form under the auspices of the local institutional review board at Hadassah Medical Organization. Confluent BMSC were used for up-regulation of CD24 experiments.

### Calculating fold increase

Calculation of CD24 fold-increase is based on the percentage of CD24 positive fraction, as measured by flow cytometry, of either the MM or B cells incubated for 4-5 days on patients BMSCs.

### Preparation of samples for sorting experiments

For sorting experiments cells were seeded on BMSC as described in the supplementary methods. After staining for CD24 cells CD24+ population and CD24- population were gated (Figure 1) and collected separately. MM and B cells were sorted using FACSAria II flow using FACSDiva software version 6.1.3 to determine gates. The sorted cells were then assessed for their ability to form colonies, migrate and divide.

A more detailed description of the materials and methods can be found in supplementary file.

## SUPPLEMENTARY MATERIALS



## Abbreviations

MM: Multiple Myeloma
PCs: Plasma Cells
BMSC: Bone marrow stromal Cells
BM: Bone marrow
PCL: Plasma Cell Dyscrasias
PB: Peripheral Blood
