# Supplementary material for: The role of CD24 in multiple myeloma tumorigenicity and effects of the microenvironment on its expression
Source: Oncotarget. 2019 Sep 10;10(52):5480–91. doi: 10.18632/oncotarget.27190 (PMC6739209; doi:10.18632/oncotarget.27190)
Supplement: Supplementary file 1 [file oncotarget-10-5480-s001.pdf]

The role of CD24 in multiple myeloma tumorigenicity and effects of the microenvironment on its expression

SUPPLEMENTARY MATERIALS

MATERIALS AND METHODS

Cell lines

MM cell lines: HL461 (kindly donated by Charlota Sandberg, Cambridge, UK), MMM1 and NCIH929 (kindly donated by Dr. Ruben Carrasco, Dana Farber Cancer Institute, Boston), RPMI-8226, U-266 and L3231 (kindly donated by Prof Bartana Hebrew University), KMS11 (Kindly donated by Prof Ben-Yehuda, Hadassah Medical Organization). CAG and JJN3 (kindly donated by Dr Katia Beider, Hebrew University). Normal B cell lines - EBV transfected: SKW6 (kindly donated by Dr Ruben Carrasco, Dana Farber, USA) and 721.221 (kindly donated by Dr Katia Beider Hebrew University). HL60 – An acute promyelocytic leukemia cell line (CD24 negative) and LAMA84 - A chronic myeloid leukemia in blast crisis cell line (CD24 positive) (both kindly donated by Prof Ben-Yehuda, Hadassah Medical Organization). All cell lines were grown and maintained in RPMI (Thermo Fisher, USA) media supplemented with 10% Fetal Calf Serum (FCS) (Thermo Fisher, USA), L-Glutamine (29.2 ug/ml), penicillin (1000 Units/ml)/ streptomycin (10ug/ml) (Biological Industries, Israel) and incubated at 37° C, 5% CO_2. All cell lines are passaged bi-weekly to maintain good cell growth.

Generation of bone marrow stromal cells from patient's BM samples

Bone marrow stromal cells (BMSC) have been generated from the left over bone marrow (BM) samples of 194 patients assessed for the presence of MM and divided into groups according to patients stage of disease; MGUS (n = 21), SMM (n = 13), active MM (Diagnosis, n = 59), patients relapsed after treatment (Relapse, n = 47), patients who did not respond to treatment (Resistant, n = 5) and patients who have low or no detectable disease (complete remission, n = 38). BM samples from patients that were diagnosed with no evidence for a hematological disease in their BM were placed in the normal group (not MM, n = 11) as a control. All patients signed an informed consent form under the auspices of the local institutional review board at Hadassah Medical Organization.

The BM from patients' samples (2–5 ml) were mixed with 10 ml of DMEM (Thermo Fisher, USA) containing 20% fetal calf serum (FCS), 1% Glutamine (29.2 ug/ml) and 1% Pen/Strep (1000 Units/ml) and loaded carefully onto 10 ml Lymphoprep, (AXIS-SHIELD Density Gradient Media – Norway) in 50 ml tube. The tube was placed into a centrifuge for 30 min at 1500 rpm. The mononuclear layer (15–20 × 10^6 cells) was collected, washed with 10 ml complete DMEM medium, suspended in 20 ml of the same medium and then placed into T75 tissue culture flasks and incubated at 37° C with 50% of medium changed once a week until cells formed a confluent adherent layer (4–8 weeks). This protocol enables generation of BMSCs layer utilized as BM microenvironment for further experiments.

Incubation of MM and B cell lines with BMSC

5 ml of trypsin EDTA solution A (0.25% Trypsin and 0.02% EDTA in Puck's solution A, Biological Industries – Israel) was added to the confluent BMSCs layer that had been established to detach the cells and re-plate the cells into 6 well plates. A T75 flask that was 90% confluent was divided over 6 35mm wells (approximately 1 × 10^6 cells/well). Once the BMSC adhered and generated confluent layer (2–7 days), 5 × 10^5 MM (KMS11 or JJN3) or B (721.221 or SKW6) cells were seeded in 3 ml of the complete DMEM medium and incubated at 37° C and 5% CO_2 for 5 days to enable CD24 up-regulation on the MM and B cells to occur. Cells were also placed in a well without the BMSC layer as control (No BMSC). After 5 days of incubation the MM and B cells were harvested, counted with trypan blue and washed with 2 ml of Phosphate buffered saline (PBS) before staining and acquisition by flow cytometer.

Treatment of MM cells with apoptotic inducing drugs

5 × 10^5 JJN3 MM cells were treated with 10, 20 and 40 nM of Velcade (Janssen- Belgium) and incubated at 37° C, 100% humidity and 5% CO_2 for 24 and 48 Hours (Velcade). After treatment, the cells were collected and stained with CD24 FITC, Annexin V APC and PI and assessed by a flow cytometer as described below.

Antibody staining for analyses by flow cytometry

Combination of monoclonal antibodies used for each series of experiments is as follows: A**.** Evaluating CD24 expression in cell lines for: i) CD24-PE and ii) Isotype control PE. Cytoplasmic staining experiments performed using permeabilization with Dako fix and perm kit according to instructions (Dako, Denmark), B. Evaluating CXCR4 expression in cell lines: i) CD24-PE, CXCR4- APC and ii) Isotype Controls for PE and APC. C Evaluating CXCR4 expression in patients: i) CD38-FITC, CD24-PE, CD45-PerCP and CXCR4-APC. PCs were gated according to CD45 and CD38 expression and the expression of CXCR4 was assessed in the CD24+ and CD24− PCs. D. Evaluating CD38 and CD24 up-regulation in cell lines: i) CD38- FITC, CD24-PE and ii) Isotype Controls FITC and-PE. E. Confluent BMSCs were trypsinized from flasks and wash twice with PBS before stain with CD24-PE antibody. All antibodies purchased from Beckman Coulter. In each experiment 1×10^6 MM, B cells or BMSCs or 100 ul of patient BM were incubated for 20 min at 4° C with appropriate combinations of antibody. The cells were then washed with 2 ml PBS and RBC lyzed and acquired by the flow cytometer. Acquisition carried out by flow cytometry FACSCalibur (Becton Dickinson BD - USA) equipped with a 488-nm blue laser and 633-nm red laser. Acquisition and analysis of data was performed with CellQuest Pro software version 5.2.1 (BD Biosciences).

Calculation of CD24 fold-increase is based on the percent of CD24 positive fraction, as measured by flow cytometry, of either the MM or B cells incubated for 4–5 days on patients BMSCs. The isotype control background auto-fluorescence is subtracted from all samples. The fold-increase in CD24 expression is calculated by dividing the detected CD24+ cells analyzed in the cells that were incubated for 4-5 days on BMSCs with CD24+ cells in cells incubated without BMSC (control).

$$\text{Fold Increase} = \frac{(\text{CD24 positive} - \text{Isotype control})\ \text{cells incubated with BMSCs}}{(\text{CD24 epositive} - \text{Isotype control})\ \text{cells incubated without BMSCs}}$$

Staining and analysis of CD24 expression on patients' samples

Combination of monoclonal antibodies used for patient samples

1) CD38- FITC, CD24-PE, CD45-PerCP, CD138-APC.

2) CD38- FITC, CD24-PE, CD45-PerCP, CXCR4-APC.

For each bone marrow sample from patients 100 µl of whole BM was incubated with 5 µl (2.5 µg) of required conjugated antibody (Beckman Coulter – France/ BD Pharmingen-USA) for 20 min at 4°C. The red blood cells were lysed with commercially available BD FACS lysing solution for 5 minutes at room temperature then washed with 2 ml PBS and resuspended in 300 µl PBS for flow cytometry acquisition. 3000 CD38 bright PCs were acquired for each tube for each patient. Assessment for percent CD45 and CD24 expression was calculated on gated CD38+CD138+ PCs. CD24 expression was assessed on PCs from patients with no disease - Not MM (n = 20), MGUS patients (n = 37), MM patients with more than 50% of PCs expressing CD45 (n = 12) and MM patients with less than 50% of PCs expressing CD45 (n = 10).

Assessment for MFI for CXCR4 was calculated on gated CD38bright PCs. 8 patient samples at diagnosis was assessed for this section of the results.

Separation of 'non-adherent' and 'adherent' cells

The incubation of MM and B cells with BMSCs generated two fractions of cells: cells that adhered to the BMSC or 'adherent' cells and cells that remained in the supernatant or 'non-adherent' cells. While harvesting the cells, the 'non-adherent' cells in the supernatant were collected first, and then the 'adherent' cells were gently released by pipetting with PBS and collected to a separate tube. The cells were stained and analyzed by flow cytometry as described above.

Conditional medium

Conditional medium (CM) was generated from supernatant collected after five days of incubation with confluent BMSC. This CM contains cytokines chemokines and growth factors released by the BMSCs. 5×10^5 MM or B cells were seeded in 3 ml of CM in one well of a 6 wells plate. After 5 days of incubation the cells were stained for CD24 expression and analyzed as described above.

RNA extraction

RNA extraction was performed with the 'total RNA mini kit' for cell culture (Geneaid, Taiwan) according to the manufacturer's instructions. Briefly, 5×10^6 MM (HL461, L3231, MMM1, NCIH929, RPM1-8226, U-2661, CAG, KMS11, JJN3) B (SKW6 and 721.221), HL60 (negative control), LAMA84 (positive control) cell lines were washed twice with PBS, resuspended in 400 µl RB Buffer containing 4 µl of β-Mercaptoethanol and incubated 5 min at room temperature to enable cell lysis. The samples were then suspended with 70% ethanol and loaded onto RB Column, placed in a 2 ml Collection Tube to discard the fluids and enable RNA binding to the column membrane. Washing the RNA sample was performed by adding 400 µl W1 buffer followed by two washes with 600 µl of Wash Buffer. The column was then placed in a new clean 1.5 ml micro centrifuge tube and 30 µl of RNase-free water were added for 2 minutes at room temperature and then centrifuged to enable RNA

elution. Before the washing step, a DNAse treatment was carried out to remove DNA traces from the RNA extraction. DNAse (5 µl, 2 U/ul) in 45 µl buffer was added to the sample and incubated 15 min at room temperature. The final RNA concentration was measured by Nano drop Spectrophotometer (Thermo Scientific- USA) using ND 1000 software version 3.7. RNA samples were stored at −80° C.

Generation of cDNA

Synthesis of cDNA from RNA was performed by using qScript cDNA Synthesis Kit (Quanta Biosciences – USA). Briefly, 1ug RNA in Nuclease-free water in a final volume of 15 µl was added together with 4 µl reaction mix and 1 µl reverse transcriptase (RT), placed in a thermal cycler (Applied Biosystems 2720 Thermal Cycler 96 well) and programmed as follows: 1 cycle at 22° C for 5 min then 1 cycle at 42° C for 30 min, to enable activation of RT, followed by 1 cycle at 85° C for 5 min to inactivate RT then cooled to 4° C. cDNA samples were stored at −20° C.

Quantitative – real time -polymerase chain reaction (q-RT-PCR)

To quantify the CD24 mRNA in the cDNA samples, CD24 Taqman probe was purchased from Applied Biosystems (Thermo Fisher Scientific – USA). HPRT (a housekeeping gene) Taqman probe was used as a control gene. A mix of 10 µl final volume containing 1 µl cDNA (25ng cDNA), 1 µl probe (CD24 or HPRT), 5 µl of fast mix buffer and 3 µl Nuclease-free water (11 µl Nuclease-free water in the negative control sample) was prepared in micro-amp tubes (Applied Biosystems). The Real-Time PCR system (StepOnePluse - Applied Biosystems) was programmed to 40 cycles of 20 sec in 95° C to enable denaturation of primers and 20 sec in 60° C to enable annealing and elongation.

Taqman probe contains reporter fluorophore at the 5′ end and a quencher at the 3′end. Increasing fluorescence indicates polymerization and an increase in the product and can be measured by the instrument while crossing the baseline threshold. Quantitation was based on the equation: Relative expression = 2-ΔΔCT by using the CT value presented in the StepOne software (version 2.2.2)

Preparation of samples for sorting experiments

For sorting experiments, 4×10^5 cells were seeded on BMSC, grown in T75 flask and treated as described previously. After staining cells were resuspended in 0.5% FCS PBS. CD24+ population and CD24− population were gated (Figure 1) and collected into tubes containing 2 ml sterile 0.5% FCS DMEM with 1.2ul/ml gentamycin (Teva –Israel). MM and B cells were sorted for CD24 expression as compared to isotype control and to cells incubated without BMSC using FACSAria II flow cytometer (Becton Dickinson (BD) – USA) equipped with a 488-nm blue laser, 630-nm red laser and 407-nm violet laser, using FACSDiva software version 6.1.3 to determine gates. MM and B cells were sorted for CD24 expression as compared to isotype control and to cells incubated without BMSC using FACSAria II flow cytometer (Becton Dickinson (BD) – USA) equipped with a 488-nm blue laser, 630-nm red laser and 407-nm violet laser, using FACSDiva software version 6.1.3 to determine gates.

Colonies formation assay

To evaluate the ability of the sorted CD24+ and CD24− cells to generate colonies in a methylcellulose based assay, 2000 sorted CD24+ or CD24− MM or B cells were seeded in each well of 24 wells plate, in duplicates, in 400µl of complete MethoCult Stem Cell Technologies – Canada). After 10 days of incubation in 37° C, 100% humidity and 5% CO_2, the numbers of the colonies generated were quantitated by counting manually under light microscope.

Migration assay

To assess the ability of CD24 + and CD24− sorted cells to migrate, 50,000 CD24+ or CD24− MM or B sorted cells were seeded in 50 µl DMEM medium containing low levels of FCS (0.5%) and placed in the upper section of a transwell System (Corning - USA). in the lower section of the well 600 µl DMEM containing high concentrations of FCS (20%) was plated. The transwell was incubated overnight in 37° C, 100% humidity and 5% CO_2 to allow the cells to migrate to the lower section via an 8-um pores in polycarbonate membrane present at the bottom of the upper chamber. The cells in the lower well were collected and percentage of cells migrating was calculated using a hemocytometer.

Cell cycle analysis

A re-suspended pellet of 2×10^5 sorted CD24+ or CD24− MM or B cells were incubated overnight at 4° C in 2 ml of 100% ethanol (AR. Gadot - Israel) for fixation purposes and to minimize clumping. The cells were then washed with 2 ml PBS and treated with 50 µl of 100 µg/ml RNase A (Sigma-Aldrich – USA) for 30 min in 37° C to remove RNA traces from the samples, then the cells were washed in PBS and 25 µl of Propidium Iodide (PI) were added. Samples were acquired and gated on FL2-Width FL2-Area to identify clumps and doublets. 10,000 events were acquired and apoptotic cells were detected in the 'SUB G1' area of graph.

For patient samples CD24-FITC and CD38-APC were used to identify the PCs as described above and then fixed and permeabilized using Dako kit Intrastain and PI was added at the permeabilization stage. Samples were acquired and gated on FL2-Width FL2-Area to identify

clumps and doublets. 200,000 events were acquired and apoptotic cells were detected in the 'SUB G1' area of graph.

Apoptosis versus necrosis

To determine if the cells up-regulating CD24 expression, post BMSC incubation, were undergoing apoptosis, cells were stained as following: 1. CD24, as described previously 2. Annexin V-APC, (BD Pharmingen-USA), that binds to phosphatidylserine that translocated from the inner side of the membrane to the outer side during apoptosis process 3. Propidium Iodide (PI) 1mg/ml (Sigma-Aldrich – USA) a DNA staining dye that can cross the membrane of dying cells only. After the cells were stained with CD24, the cells were wash with PBS and re-suspended in 100 µl of Annexin V binding buffer (BD Pharmingen USA) and incubated with 5 µl Annexin V - APC for 15 min at room temperature. The cells were then washed in binding buffer, re-suspended in fresh 400 µl Binding Buffer and 2 µl PI was added. After incubation of 5 min the samples were analyzed by flow cytometry. Unstained sample, sample with Annexin V –APC with and without binding buffer and samples with PI alone were used as controls for gating and compensation. For analysis, the cells were gated by CD24+ and CD24−expression and the percentage of Annexin V/ PI positive cells were calculated in each fraction.

Statistics

All *in-vitro* experiments were performed at least in triplicate and repeated at least 3 times. All figures are an average of at least 3 experiments. Statistical significance of differences was determined using Students t test, with minimal level of significance $p<0.05$. All statistical analyses were determined using Microsoft Excel software. Data presented as standard error mean (SEM) of the results in each experiment.

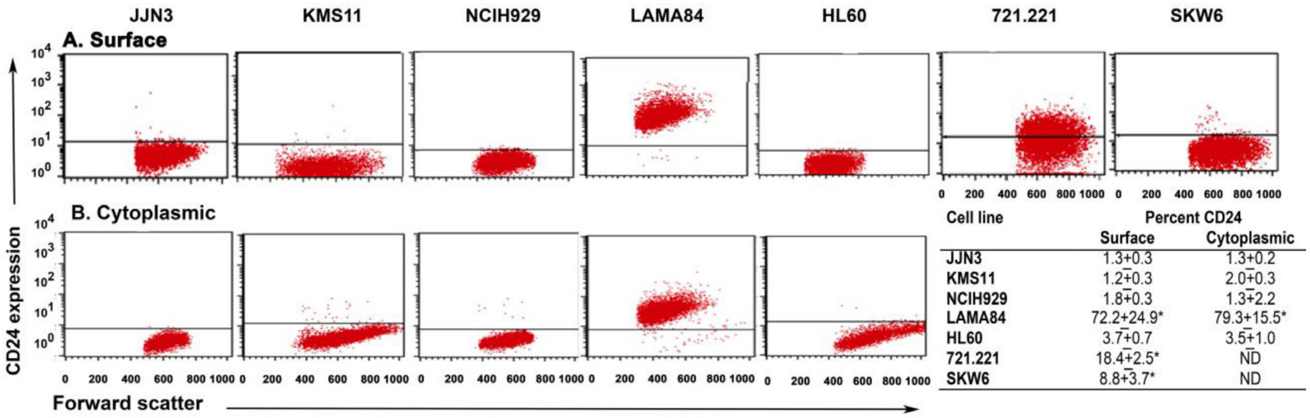

Cell line	Percent CD24 Surface	Percent CD24 Cytoplasmic
JJN3	1.3±0.3	1.3±0.2
KMS11	1.2±0.3	2.0±0.3
NCIH929	1.8±0.3	1.3±2.2
LAMA84	72.2±24.9*	79.3±15.5*
HL60	3.7±0.7	3.5±1.0
721.221	18.4±2.5*	ND
SKW6	8.8±3.7*	ND

Supplementary Figure 1: Surface and cytoplasmic expression of CD24 in MM and B cell lines. Representative dot plots of CD24 expression in MM cell lines; JJN3, KMS11, NCIH929; Positive control = LAMA84; Negative control = HL60 and B cell lines; SKW6 and 721.221. (**A**) Surface staining, (**B**) Cytoplasmic staining and (**C**) Mean ± SD percent of CD24 expression, n = 3, *denotes significant differences compared to HL60.

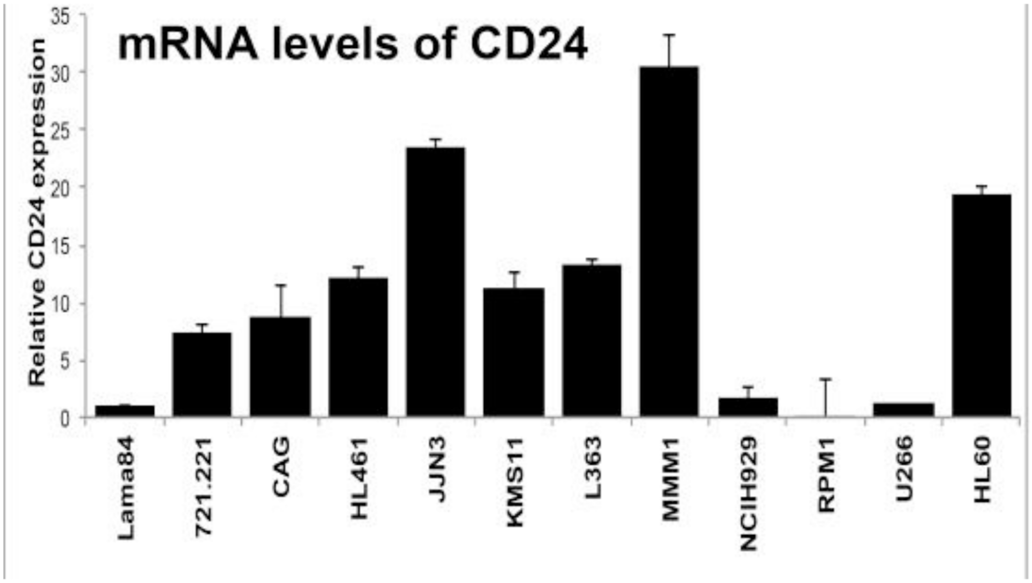

Supplementary Figure 2: mRNA expression of CD24 in cell lines. Relative expression of CD24 mRNA in LAMA-84 - used as positive control, 721.221 a B cell lines, various MM cell lines (CAG, HL461, JJN3, KMS11, L363, MMM1, NCIH929, RPM1, U2661) and HL60 – used as negative control as quantified by q-RT-PCR. HPRT was used as endogenous control for the reaction. The expression in all samples is relative to LAMA84 (n = 3).

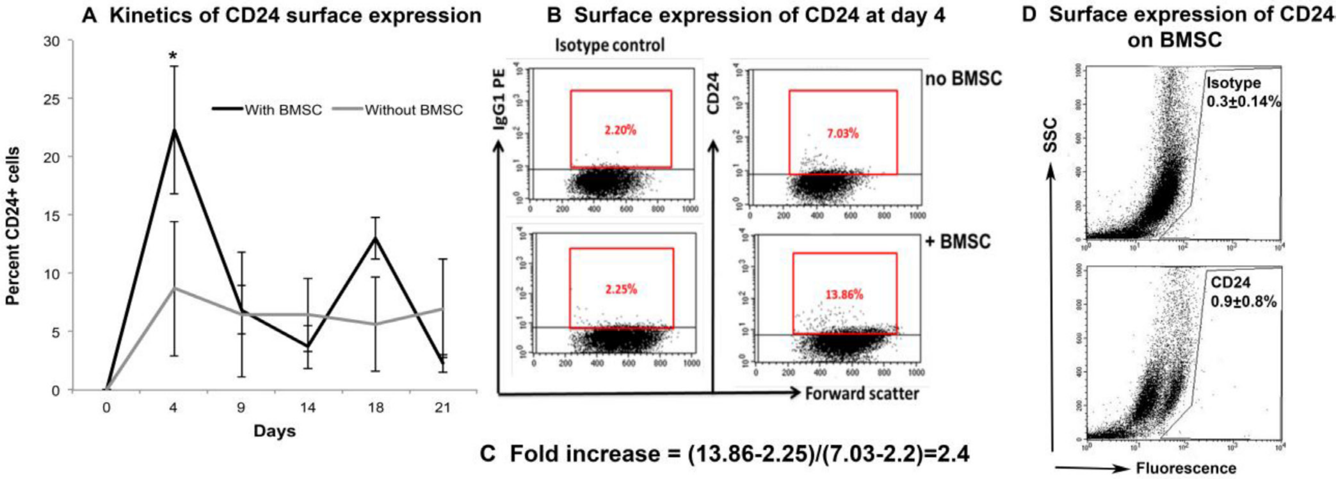

Supplementary Figure 3: BMSCs generated from MM patient can up-regulate CD24 expression after co-cultured MM cells. (**A**) MM cells (JJN3, KMS11) were incubated with (black) and without (grey) BMSC generated from patients with BMSC and harvested at various time points. The percent of CD24 expression was analyzed by flow cytometer at each time point (n = 4). (**B**) Representative dotplot of CD24 expression on JJN3 (MM) cells incubated with (+BMSC) or without (no BMSC) BMSC for five days. (**C**) Formula used for calculating fold increase in CD24 expression. Fold increase = (%CD24+ cells after incubation with BMSC - % isotype control background)/%CD24+ cells with incubation on BMSC - % isotype background) for each individual BMSC generated and incubated either with MM or B cell lines. (**D**) CD24 expression was assessed on BMSC grown from MM patients (mean ± SD, n = 5) *denotes significant difference to no BMSC

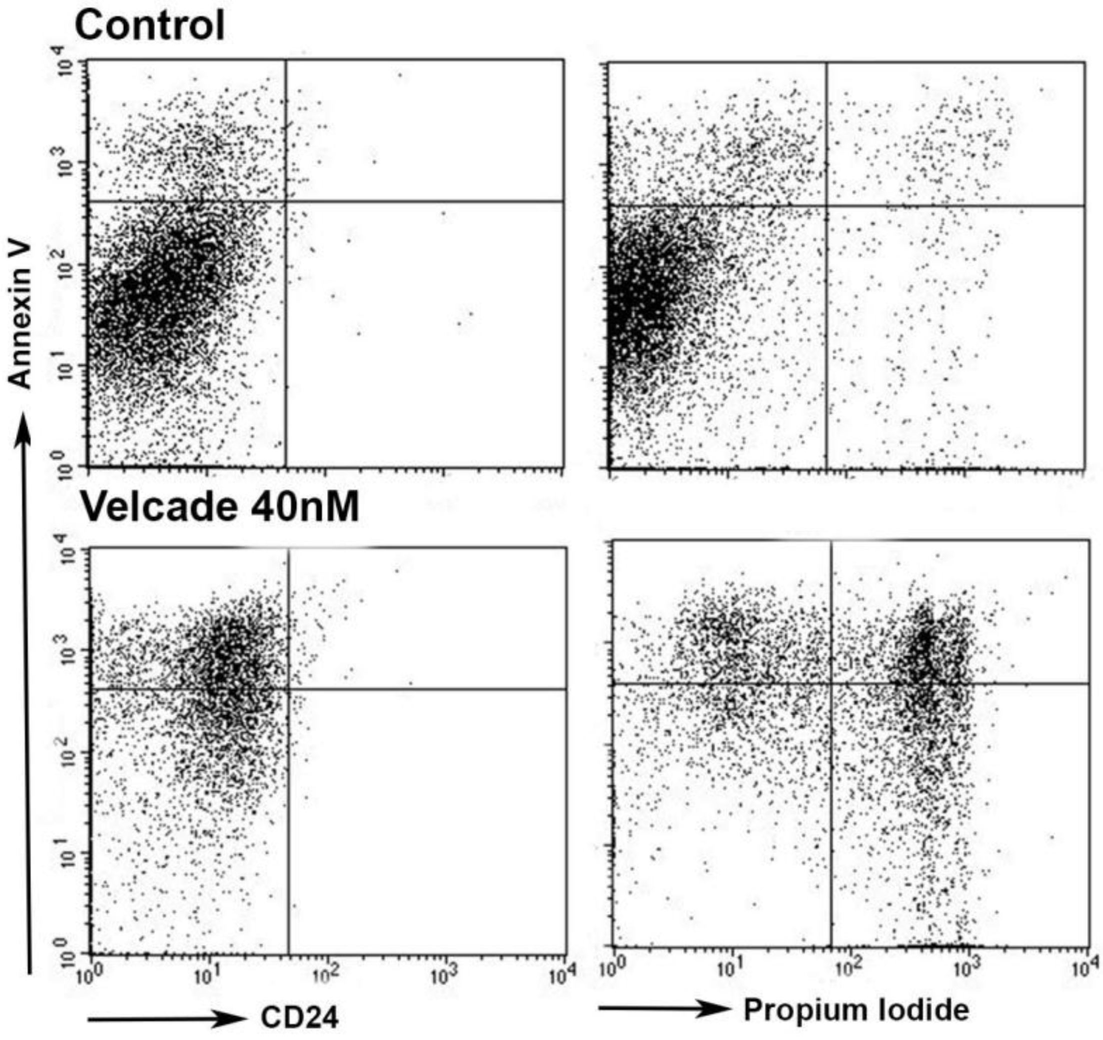

	AnnV$^+$CD24$^+$	PI$^+$CD24$^+$	AnnV$^+$PI$^+$	AnnV$^+$PI$^-$
**No Velcade**	0.25±0.18	0.10±0.09	2.86±1.04	9.61±3.07
**10nM Velcade**	0.80±0.71	0.47±0.62	27.24±1.55*	39.92±9.21*
**20nM velcade**	0.31±0.73	0.24±0.38	31.56±11.16*	30.07±9.20*
**40nM velcade**	0.55±0.25	0.28±0.26	33.83±11.94*	34.33±1.64*

Supplementary Figure 4: Velcade induction of apoptosis does not increase CD24 expression on the surface of MM cells. MM cell line was incubated with no Velcade or 10, 20 and 40 nM of Velcade and assessed by flow cytometry for apoptosis (Annexin V) necrosis (propidium iodide) and CD24 up-regulation after 48 hours. Significant increase in Annexin V and Propidium Iodide (PI) double positive cells with were detected in the cultures however no increase in CD24 was found (*n* = 5).

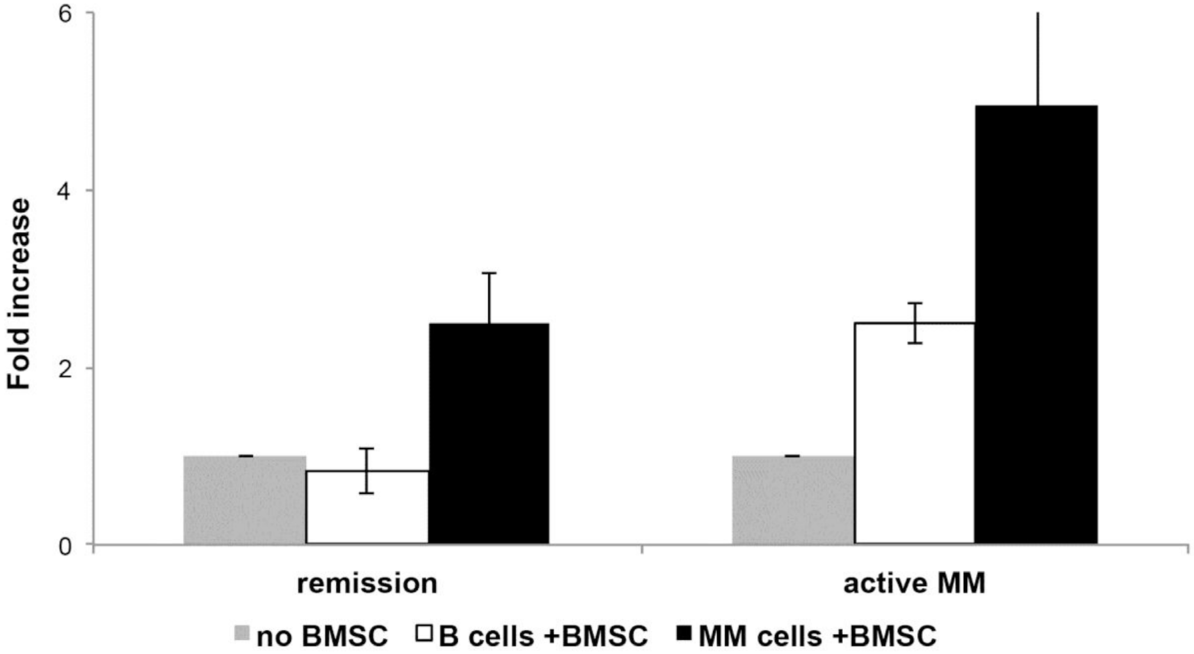

Supplementary Figure 5: The effect of MM progression on CD24 up-regulation on MM and B cell lines. Fold increase in CD24 expression in B ((remission *n* = 5 and Active MM *n* = 13) or MM (Not MM *n* = 6, Inactive MM *n* = 10, Remission MM *n* = 9 and Active MM *n* = 22) cells incubated with BMSCs over B or MM cells incubated without BMSCs. (mean ± SEM).
